# Left Compartmentectomy for Adult-Type Granulosa Tumor of the Retroperitoneum: A Case Report and Review of the Literature

**DOI:** 10.7759/cureus.75867

**Published:** 2024-12-17

**Authors:** Moctar Noufou Fodiya, Ali Kamil Mohamed, Zakaria Elmouatassim, Habib Dato Outban, Ayee Afetane Stéphane Arnaud, Kaid Mohamed Kaid, Omar Belkouchi, Zouaidia Fouad, Hadj Omar El Malki

**Affiliations:** 1 Surgery A, Moulay Abdellah Hospital, Salé, MAR; 2 General Surgery, Faculty of Medicine and Pharmacy, Mohamed V University, Rabat, MAR; 3 Anatomical Pathology, Ibn Sina Hospital, Rabat, MAR; 4 Pathological Anatomy, Faculty of Medicine and Pharmacy, Mohamed V University, Rabat, MAR; 5 Clinical Research and Epidemiology, Mohamed V University, Rabat, MAR

**Keywords:** adult granulosa tumors, compartmentectomy, extra-ovarian localisation, immunohistochemistry, retroperitoneal tumors

## Abstract

Granulosa tumors are rare tumors arising from the cells of the sexual cord and stroma of the ovary. They account for 5% of ovarian cancers and 70% of stromal cancers of the sex cords. Retroperitoneal tumors (RPTs) are also rare and develop in the retroperitoneal and subperitoneal space. They are usually diagnosed late and are dominated by sarcomas and around 10% of teratomas. Based on principles derived from limb sarcoma surgery, compartmentectomy is currently the approach recommended by experts for the surgical removal of soft tissue sarcomas of the retroperitoneum. A primary retroperitoneal tumor composed of granulosa cells arising away from the ovaries, which is the usual location, is extremely rare.

We describe a case of a left compartmental excision, removing the kidney and homolateral ureter, the left colon enlarged to the transverse with colorectal anastomosis for retroperitoneal granulosa tumor of the adult type in a 40-year-old woman.

## Introduction

Granulosa tumors are the most frequent ovarian malignancies in the group of sexual cord and stromal tumors. However, their extra-ovarian localization is rare and retroperitoneal localization is exceptional [1]. Retroperitoneal tumors are often enormous and may invade neighboring organs, implying a radical surgical approach with the possibility of multi-organ resection. We report the case of a left compartmentectomy for an adult-type granulosa tumor of the retroperitoneum in a 40-year-old woman for its rarity while sharing our experience regarding diagnostic and therapeutic difficulties.

## Case presentation

**Figure 1:**
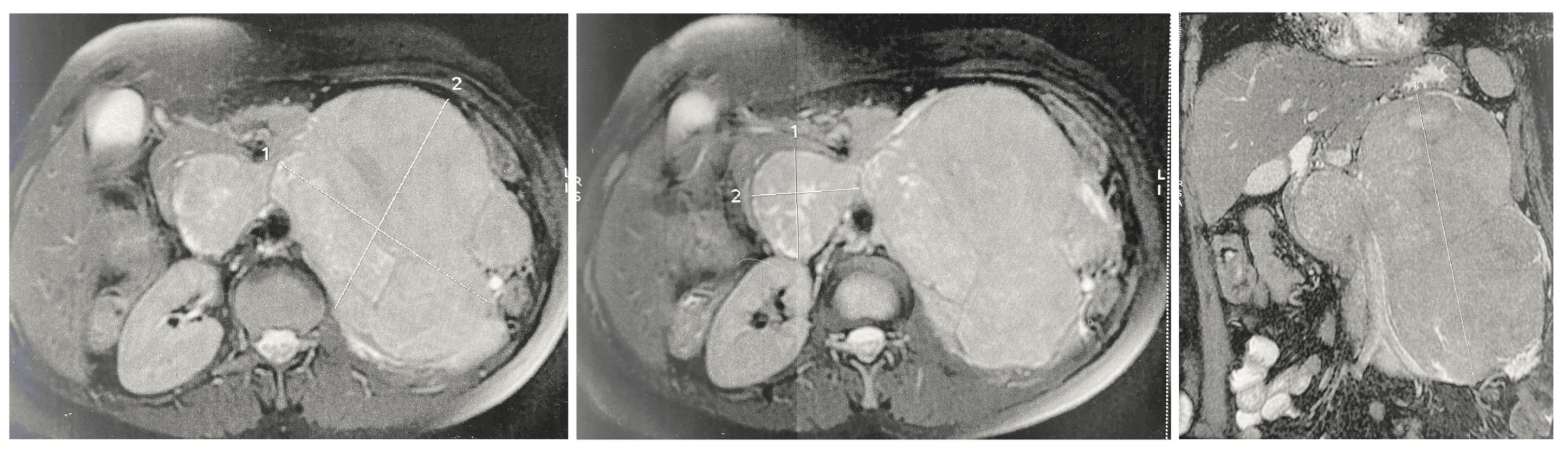
Abdominal-pelvic MRI images showing the dimensions of the mass (215 x 122 x 177 mm) and its intimate relationship with the aorta, left kidney and left urethra

This is the case of a 40-year-old woman, nulligravida with no known medical history but with antecedent abdominal surgery in 2019 reported by the patient. We have no proof of this surgery (no documents). In January 2024, a retroperitoneal mass was discovered during a follow-up scan. She was referred to our facility for surgical management. On admission, she was asthenic, apyretic, malnourished with a body mass index of 17 m²/kg, blood pressure of 90/50 mmHg, heart rate of 60 beats per minute, and oxygen saturation of 100% in room air. Abdominal examination revealed a distended abdomen, with diffuse tenderness to palpation. The biological tests ordered were all normal, and the most important are summarized in Table 1. Abdomino-pelvic CT showed a large left retroperitoneal juxta-spinal, extra-adrenal and extra-renal mass pushing back neighboring structures and stretching neighboring vessels without any sign of invasion, associated with other retroperitoneal and posterior infra-mediastinal masses of similar appearance. This appearance raises suspicion of ganglioneuroma, lymphomatosis or phacomatosis. Abdominal-pelvic MRI revealed a fairly well-limited, roughly oval, polylobed, intra- and retroperitoneal left abdominal-pelvic tissue lesion process with T1 hypo signal, discrete 12 hyper-signal with diffusion restriction and antibody-drug conjugates (ADC) drop enhanced after gadolinium injection delineating large areas of necrosis measuring approximately 215 x 122 x 117 mm. This process comes into contact with the left kidney, which it pushes backwards with a safety fat border and is responsible for moderate left ureterohydronephrosis. It engulfs the left renal artery and vein around their entire circumference, with no detectable thrombosis. It also comes into contact with the left psaos muscle, with no obvious tumor infiltration. Anteriorly, it displaces the jejunal ansae and the left colon; medially, it contacts the celiac trunk, the superior mesenteric artery and the inferior mesenteric artery, with a safety fatty interface. At the bottom, it displaces the left iliac pedicle, which it engages at an angle of less than 180°. Peri-injury adenopathies are also present, the most voluminous of which are located and measured: Right latero-aortic adenopathy adjoining the duodenum measuring 52x53 mm, retro-cava adenopathy measuring 37x23 mm, and internal iliac adenopathy measuring 44x39 mm. Uterus seen with normal appearance but ovaries not seen (Figure 1). Thoracic CT showed no secondary intrathoracic location. A scan-guided biopsy was performed, with anatomopathological and immunohistochemical analysis in favor of a ring-tubule sex cord tumor or granulosa tumor.

**Table 1:** Biological analysis CRP: C-reactive protein; CA 125: cancer antigen 125; CEA: carcinoembryonic antigen; CA 19-9: carbohydrate antigen 19-9; PT: prothrombin time

Analysis	Values obtained	Reference values	Unit
Hemoglobin	12.2	12-16	g/dl
Platelets	331,000	150,000-400,000	/mm³
PT	93	>70	%
CRP	11	<6	mg/l
CEA	0.56	<4.7	ng/ml
CA 125	10.06	<35	U/ml
CA 19-9	13.86	<37	Ul/ml

**Figure 2:**
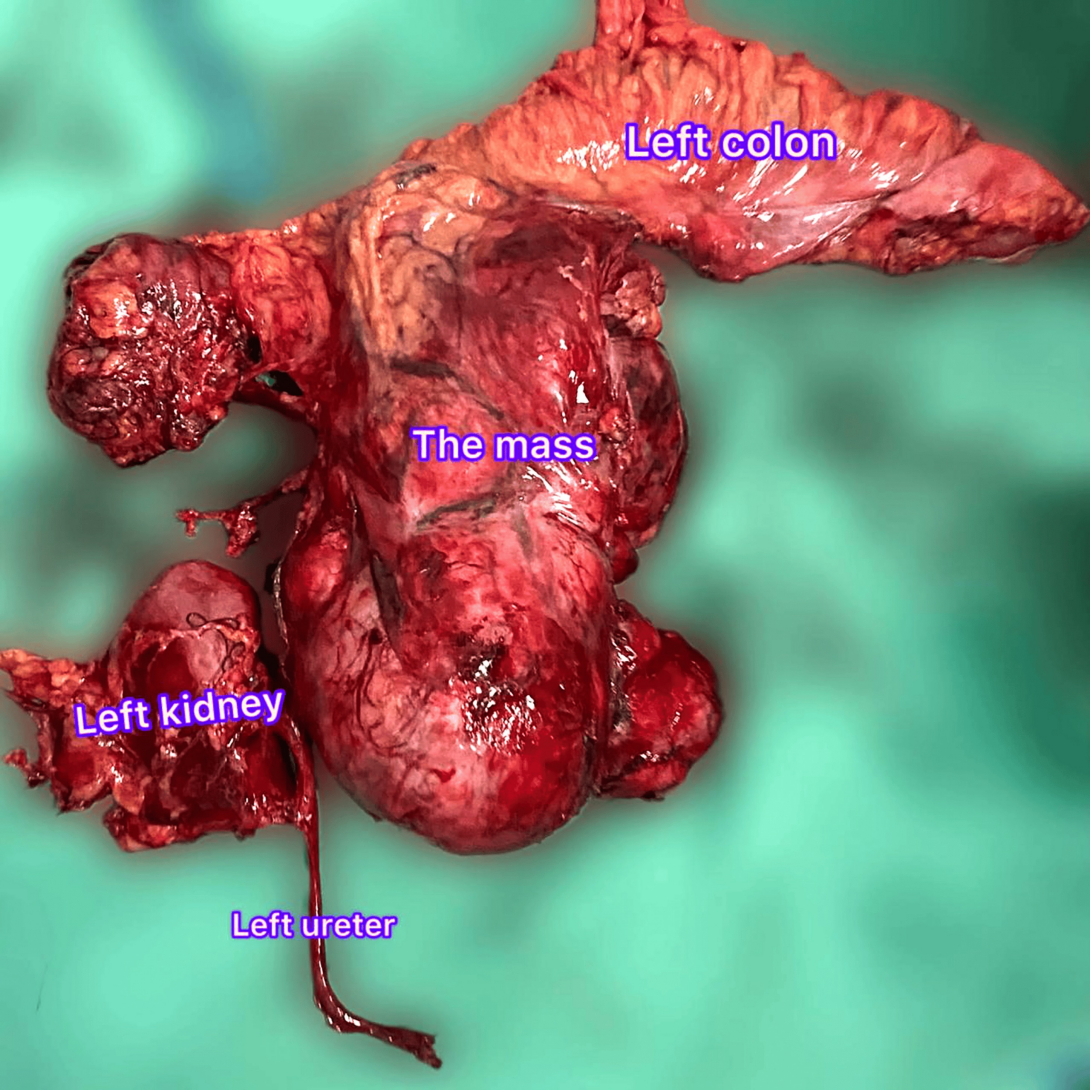
Surgical specimen showing monobloc mass with left colon, left kidney and left ureter

An indication for surgery was given in view of the voluminous appearance of the mass, with compression of the neighboring organs. The patient was taken to the operating room. Intraoperative exploration revealed a large bilobed retroperitoneal mass coming into contact with the duodeno-pancreatic framework and pushing back the left colon. We started with a left colo-parietal approach, freeing the mass from the iliac vessels, then proceeded with a right-to-left colo-epiploic detachment to access the back cavity of the epiploons. We freed the mass from the duodeno-pancreatic framework, the inferior vena cava and the inferior base of the pancreas. After control and ligation of the left ureter, followed by control and ligation of the renal pedicle, we released the loop mass and performed a colonic section and a rectal section. This enabled us to perform a monobloc left compartmentectomy, removing the left kidney, the left ureter and the left colon enlarged at the transverse (Figure 2). We finished by performing a mechanical colorectal anastomosis. During the operation, the patient experienced episodes of hypotension, which were managed by injecting drugs. The operation lasted five hours, with an estimated blood loss of 600 ml. She was transfused with two 450 ml bags of packed red blood cells. The patient's operative specimen was sent for anatomopathological analysis. The immediate postoperative course was marked by the formation of a liquid effusion at the compartmentectomy site. Scanno-guided drainage was performed and the patient was put on antibiotic therapy with a favorable outcome.

**Figure 3:**
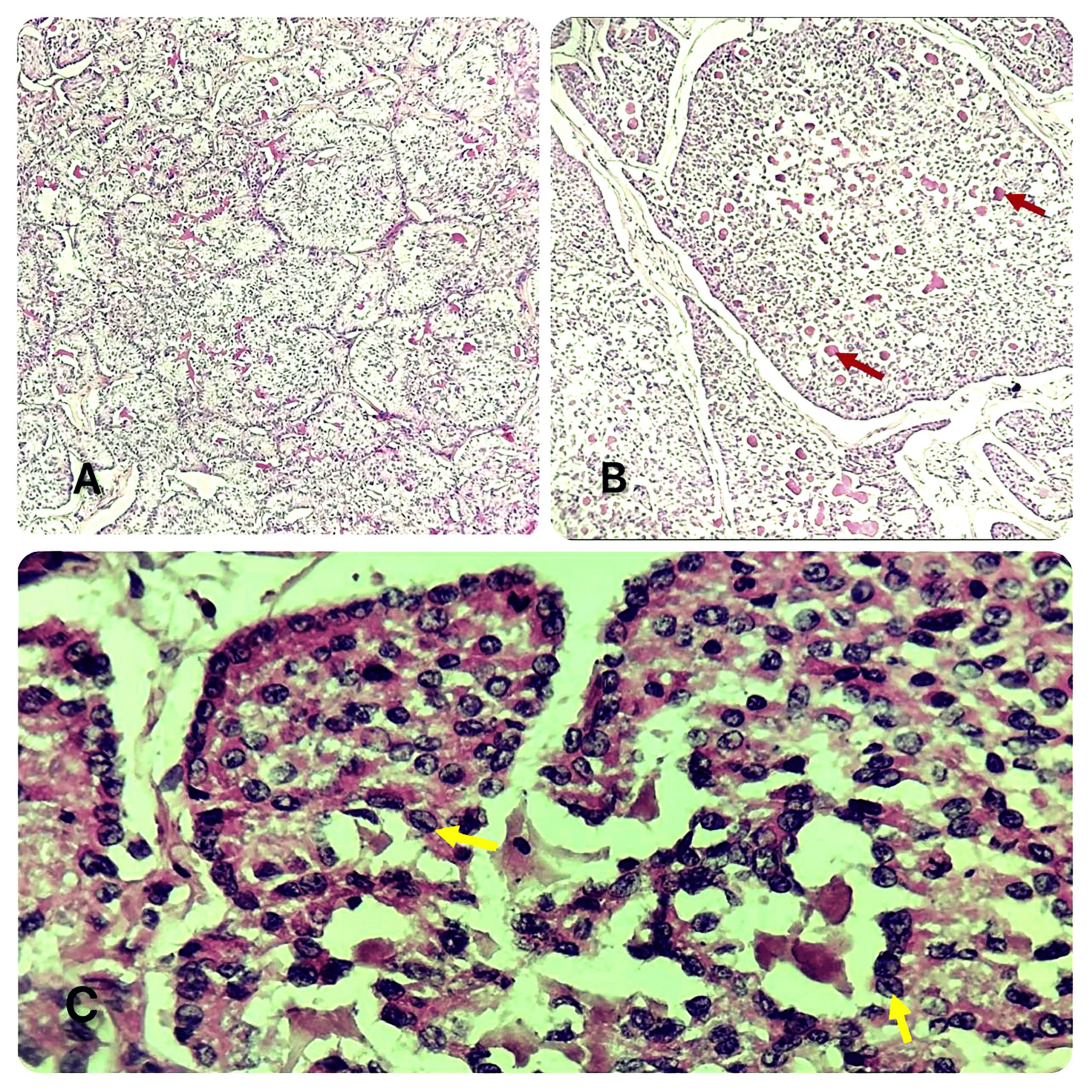
Pathological analysis images Image A: microscopic appearance at low magnification showing tumor proliferation with microfolllicular, solid, trabecular and insular architecture (HEx100). Image B: microscopic appearance at high magnification showing Call-Exner bodies: cells arranged in rosettes of eosinophilic material, arrows in red (HEx400). Image C: cuboid tumor cells with sparse cytoplasm and globally uniform, round, angular nuclei with coffee-bean grooves, yellow arrows.

Anatomopathological examination of the various samples taken from the macroscopically identified mass shows tumor proliferation of variable architecture from one area to another. They range from micro-follicular masses with eosinophilic content (Call and Exner bodies) to lobules, trabeculae or solid masses. Tumor cells have abundant eosinophilic or clear cytoplasm. Nuclei are round or oval, sometimes with visible nucleoli. The stroma is loose and vascular. Samples from two nodules found macroscopically correspond to the same histological description described above. Morphological appearance was suggestive of an adult granulosa tumor with two nodules of carcinosis (Figure 3). The ureteral and colonic borders were healthy. The kidney and colon were free of tumor infiltration. Lymph node dissection was negative.

## Discussion

Granulosa tumors are the most frequent malignant tumors in the group of sex cord and stromal tumors. They account for 5% of ovarian cancers and 70% of stromal cancers of the sex cords [2]. Two types are distinguished: juvenile and adult. The adult form is the most frequent and occurs most often in the premenopausal period or early menopause, with a median age of 50-54 years [3]. Apart from the most common ovarian localization, other forms of localization have been reported in the literature. They can develop in the broad ligament, retroperitoneum, mesentery, liver, adrenal glands, omentum and fallopian tubes [3,4]. The origin of extra-ovarian forms is poorly understood, but it is assumed that they arise from ectopic gonadal tissue along the embryonic genital ridge pathway [5].

Retroperitoneal localization is rare, in fact, after a meticulous search of the literature using the keywords “adult granulosa tumour”, “extra-ovarian” and “retroperitoneal”, only 16 cases were reported [4,6-9]. In addition to being in the menopausal period (40 years) and nulligravida (a factor favoring early menopause), our patient's history may corroborate the occurrence of a tumor type. A history of oophorectomy in women is a risk factor for the development of extra-ovarian giant cell tumors such as those of the retroperitoneum [3,9]. Non-visualization of the ovaries on magnetic resonance imaging and a history of undocumented surgery may suggest previous ovarian surgery. Abdominal distension is the clinical sign most commonly reported in retroperitoneal forms. This distention may or may not be accompanied by pain. In our case, distension was accompanied by abdominal pain. The granulosa tumor of the retroperitoneum appears as a large tumor in the cases reported, whose dimension was mentioned. Dimensions ranged from 6-21 cm long axis. Ours is the second largest mass reported with 21.5x12.2x11.7 cm, behind 21x20cm reported by Menon et al. [7].

The difficulty of preoperative diagnosis lies in the fact that radiological signs are not specific, and radioguided biopsy is not obvious because of its location. In our case, CT and MRI scans enabled us to describe the size of the mass and its relationship with neighboring organs. We were able to perform a CT biopsy, but it was not specific, as on immunohistochemistry, there was doubt between a ring-tubule sex cord tumor and a granulosa tumor. Diagnosis of the disease is based on anatomopathological examination. The histological form of granulosa tumors of the retroperitoneum is similar to that of normal granulosa. Tumor cells have uniform, round or oval nuclei with finely granular chromatin and longitudinal nuclear grooves or folds [3].

Surgery is the first-line treatment for granulosa tumors. Surgery to remove tumors of the retroperitoneum meets carcinological imperatives in terms of quality of excision and technical requirements in terms of resectability [10]. Compartmental excision or compartmentectomy, whose principles derive from limb sarcoma surgery, which has a true compartment, is the recommended approach for improving oncological results in retroperitoneal tumors. This technique is most frequently used in retroperitoneal sarcomas. It consists of a monobloc resection of the tumor and underlying organs. It has been standardized and is currently the approach recommended by leading expert groups [11-14]. The team at the Memorial Sloan Kettering Cancer Center in New York suggests extending en bloc excision to organs adjacent to the tumor, even when they are not invaded, in order to obtain healthy margins [15]. The therapeutic difficulty we were faced with stemmed from the fact that we had no preoperative histological diagnosis. Given that sarcomas are the most common retroperitoneal tumors, we opted for a radical approach to minimize the risk of recurrence. With this in mind, we performed a complete monobloc resection by left compartmentectomy in our patient, removing the left kidney, the left ureter and the left colon extended to the transverse colon (Figure 3). Chemotherapy is considered for more advanced forms that cannot be surgically resected. Adjuvant chemotherapy should include platinum-based therapy, either carboplatin and paclitaxel, etoposide and cisplatin (EP), or bleomycin, etoposide and cisplatin (BEP) [6]. Our patient was referred to the oncologists after a multidisciplinary meeting for further management on discharge.

The prognosis of the disease depends on a number of factors, including the patient's age: in fact, an age under 40 is a good prognostic factor. As regards tumor size, a tumor larger than 10 cm is correlated with an increased risk of recurrence. With regard to stage, an advanced stage is predictive of recurrence. Other factors include tumor rupture, nuclear atypia, ploidy and cell proliferation index, p53 mutations, etc. [16]. The first three factors are present in our patient, which means that there is a risk of recurrence. Close monitoring is required to detect any recurrence.

## Conclusions

A primary retroperitoneal tumor composed of granulosa cells and developing far from its usual location (in the ovary) is less frequently observed. Although anatomopathological examination remains the key to diagnosis, it must be suspected in women who have undergone oophorectomy. Wide and complete surgical resection (compartmental resection) should be considered.
